# Out-of-plane performance of structurally and energy retrofitted masonry walls: geopolymer versus cement-based textile-reinforced mortar combined with thermal insulation

**DOI:** 10.12688/openreseurope.16724.1

**Published:** 2023-10-30

**Authors:** Szymon Cholostiakow, Ioanna Skyrianou, Lampros Koutas, Christos Papakonstantinou

**Affiliations:** 1 Department of Civil Engineering, University of Thessaly, Volos, Greece

**Keywords:** seismic strengthening, energy retrofitting, masonry, masonry infills, reinforced concrete, textile reinforced mortar, TRM, geopolymers, alkali-activated materials

## Abstract

This paper examines the out-of-plane performance of masonry walls (representative of infills in reinforced concrete frames) which have been upgraded with an outer skin of integrated structural and an energy retrofitting system. The benefits of such an integrated system are mainly cost-related. Nevertheless, before moving to full-scale applications, additional benefits to the structural performance need to be investigated. In this study, the examined configurations of this composite system comprised either thermal insulation boards bonded directly to the wall followed by layers of textile-reinforced mortar (TRM), or thermal insulation boards bonded in-between two TRM layers. Other than the retrofitting layers configuration, the following parameters were also investigated: a) the binder type (cement-based versus geopolymer-based mortars), and b) the textile type (open mesh glass fibre textile versus basalt fibre textile). The results of this experimental study are discussed in terms of failure modes, post-cracking stiffness and ultimate capacities. Overall, this study highlights the mechanical benefits of the TRM plus thermal insulation system while providing insights on the bond performance between the different materials selected. An important finding is that the integrated system is even more effective than a standard TRM application. Finally, the geopolymer mortar seems to be equivalent in terms of performance to the commercially available cement-based mortars.

## Plain language summary

The research presented herein deals with novel composite materials for structurally and energy deficient masonry buildings. The paper offers practical insights into integration of standard energy retrofitting and structural retrofitting using innovative and sustainable materials such as geopolymer mortars reinforced with basalt or glass textiles. Geopolymers, which belong to the family of alkali-activated materials (AAM), are innovative inorganic polymers, which can be used as binding materials in construction. Geopolymer-based mortars have the potential to reduce the construction sector’s CO _2_ emissions by replacing Ordinary Portland Cement (OPC). Such mortars can be used as binders for open mesh textiles and their production is associated with less CO _2_ emissions compared to OPC-based binders. The use of low-cost basalt and glass textiles allows for good balance between cost and efficiency. Such advanced composite systems combined with thermal insulation and applied to the envelopes of buildings can tackle both structural and energy deficiencies and yet offer a low carbon footprint at a reasonable cost.

## Introduction

Unreinforced masonry (URM) is the typical material used for infilling reinforced concrete (RC) frames in southern Europe and other areas worldwide. The presence of URM infills, however, significantly affects the seismic performance of the buildings ( Dias-Oliveira *et al.*, 2022; Fardis & Panagiotakos, 1997; Mehrabi *et al.*, 1996; Morandi *et al.*, 2022; Zarnic & Tomazevic, 1988). The brittle nature of such walls often leads to premature failures, thus making them vulnerable if proper measures are not taken at the initial design stage. The assessments of past major earthquakes highlighted various damages associated with URM infills, with partial or global out-of-plane (OOP) collapse of the infill walls being the dominant failure mode ( Augenti & Parisi, 2010; Furtado *et al.*, 2021c; Manfredi *et al.*, 2014; Sezen *et al.*, 2003). Thus, structural retrofitting of such walls is in many cases deemed necessary to upgrade seismic performance and improve the safety of existing buildings ( Gkournelos *et al.*, 2021). The most vulnerable are buildings of the 90’s era and back, built using limited seismic provisions and often lacking proper detailing against lateral loads. In addition, these structures have been suffering for decades from high energy consumption for heating/cooling demands, as no energy provisions were in force at the time of construction. A holistic approach addressing both structural and energy performance needs has been recently proposed ( Bournas, 2018; Gkournelos *et al.*, 2019; Triantafillou *et al.*, 2017; Triantafillou *et al.*, 2018), setting up a new direction for novel and more comprehensive retrofitting approaches for masonry structures ( Cholostiakow *et al.*, 2023b; Chrysostomou *et al.*, 2023; Furtado *et al.*, 2022; Furtado *et al.*, 2023a; Furtado *et al.*, 2023b; Gkournelos *et al.*, 2020; Karlos *et al.*, 2020). The proposed integrated retrofitting system combines conventional insulation materials such as expanded polystyrene (EPS) boards with one or more textile-reinforced mortar (TRM) layers, as such, integrating the two into one composite material and offering a system which can tackle structural and energy deficiencies simultaneously. Other alternatives have also been suggested and investigated experimentally ( Ademovic *et al.*, 2022; Baek *et al.*, 2022; Longo *et al.*, 2020; Pohoryles *et al.*, 2022).

Externally bonded TRM systems consist of a) open-mesh textiles with high-strength fibre rovings typically stitched in two orthogonal directions, and b) typical-to-high strength mortars which serve both as binders to the substrate and as matrix materials for the textile reinforcement thus enabling composite action. The beneficial effect of TRM systems on the structural performance of various types of TRM-retrofitted structural elements (both concrete and masonry) has been systematically verified by many studies ( Kouris & Triantafillou, 2018; Koutas *et al.*, 2019). Regarding structural retrofitting of masonry-infilled reinforced concrete (RC) frames, TRM jacketing has been proved to be very effective ( Akhoundi *et al.*, 2018; Akhoundi *et al.*, 2023; Da Porto *et al.*, 2015; De Risi *et al.*, 2020; De Risi *et al.*, 2022; Facconi *et al.*, 2018; Facconi & Minelli, 2020; Furtado *et al.*, 2021a; Furtado *et al.*, 2021b; Ismail *et al.*, 2018; Koutas *et al.*, 2015; Koutas & Bournas, 2019; Minotto *et al.*, 2020). The particular benefits include a) increase in the initial elastic stiffness and the in-plane capacity of the infilled frames, b) enhancement of the infills’ energy dissipation capacity, c) improvement of the out-of-plane capacity of the infills while mitigating the risk of their out-of-plane collapse, and d) delay in the development of soft storey mechanism in multi-storey frames, which ultimately reduces the risk of buildings’ total collapse during an earthquake.

Although the structural efficiency of TRM has been assessed experimentally by many research studies, the knowledge on the mechanical performance of composites with integrated TRM and EPS systems is still very limited. Most past studies have focused on the OOP flexural performance of either masonry prisms ( Furtado *et al.*, 2023a; Gkournelos *et al.*, 2020; Karlos *et al.*, 2020; Triantafillou *et al.*, 2017) or masonry infill walls in RC frames ( Gkournelos & Triantafillou, 2023). The results of these studies are very promising, though they refer to specific selection of materials and strengthening configurations.

Recent attempts to reduce the environmental impact of TRM systems focused on geopolymers as new binding materials for the textiles, which have potential to become an alternative to Ordinary Portland Cement (OPC) binders ( Arce *et al.*, 2022a; Askouni *et al.*, 2021; Cholostiakow *et al.*, 2023a; Ghiassi *et al.*, 2016; Gkournelos *et al.*, 2022; Skyrianou *et al.*, 2022; Tamburini *et al.*, 2017; Wang *et al.*, 2021; Zhang *et al.*, 2019; Zhang *et al.*, 2022). Geopolymers, which can be formed by mixing silicate dry products with an alkaline solution, exhibit excellent mechanical properties, high durability, and resistance to elevated temperatures, thus making them potential candidates as an alternative to cement-based materials, but also in a variety of uses as a fireproof adhesive for fibre-reinforced composites and laminates ( Arce *et al.*, 2022b; Chen *et al.*, 2021; Giancaspro *et al.*, 2009; Giancaspro *et al.*, 2010; Katakalos & Papakonstantinou, 2009; Mobili *et al.*, 2016; Papakonstantinou *et al.*, 2001; Papakonstantinou & Balaguru, 2006; Papakonstantinou & Balaguru, 2007; Papakonstantinou & Katakalos, 2009; Zhang *et al.*, 2018). Recent studies suggest that when geopolymer binders are used instead of OPC binders, the greenhouse gas emissions can be reduced by up to 64% and even lower production costs can be anticipated ( Komkova & Habert, 2023; McLellan *et al.*, 2011). Since the type of the mortar has proven to have strong influence on the overall performance of externally bonded TRM systems ( Koutas & Papakonstantinou, 2021), direct comparisons between cement-based and geopolymer-based TRM systems are timely and very important to assist in future developments of new geopolymer binders.

This paper examines the OOP performance of masonry walls commonly used as infills in RC frames. New experimental insights into the experimental behaviour of four standard (TRM) and eight integrated (TRM + EPS) systems are presented and discussed. The main goal of this research was to investigate the performance of a metakaolin-based geopolymer binder as a matrix for textile reinforcements used for masonry strengthening in combination with thermal insulation. Different combinations of textiles, binders and retrofitting layouts were explored to better understand the effects of these parameters on the global OOP response and failure. The main objectives of the experiments were: i) to assess how different retrofitting layouts can affect the OOP performance of such integrated composite systems, and how this compares to regular TRM strengthening without insulation; ii) to assess how different types of textiles affect the global OOP performance of the walls; and iii) to assess how different types of binders affect the global OOP performance of the walls. The results of the study are being discussed in terms of failure modes, bending capacity, stiffness and suggest the best configuration to be used in masonry-infilled RC frames.

## Methods

**Figure 1.**
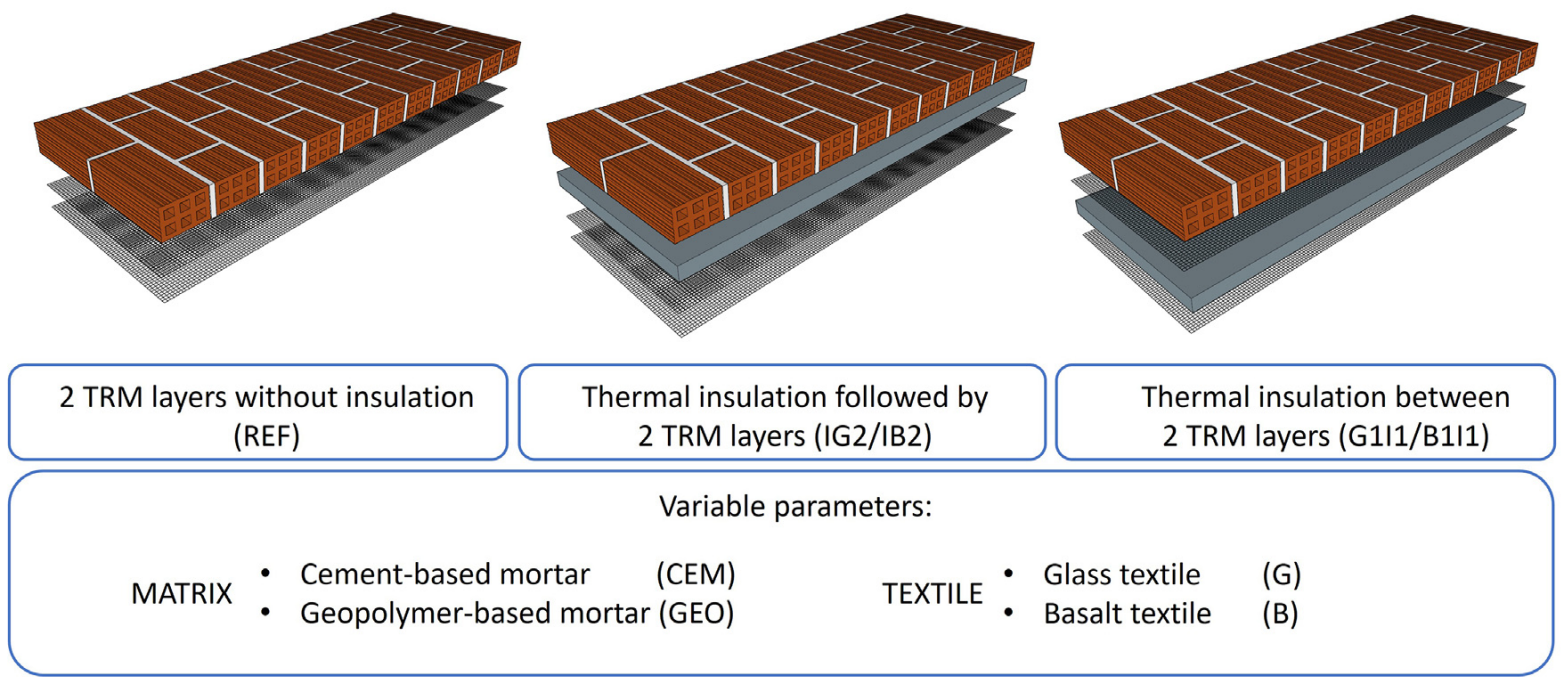
Layout of the experimental programme.

The experimental programme (see Figure 1) consisted of 12 OOP tests on masonry walls retrofitted in three different configurations: i) structural retrofitting with two TRM layers without insulation (REF); ii) combined structural and energy retrofitting with thermal insulation directly attached to the masonry surface followed by two TRM layers (IG2/IB2); iii) combined structural and energy retrofitting with thermal insulation between the two TRM layers (G1I1/B1I1). For each strengthening layout, two different matrices (geopolymer versus cement-based mortar) and two different commercial textiles (basalt versus glass-fibre mesh) were investigated.

### Specimen geometry and notation

**Figure 2.**
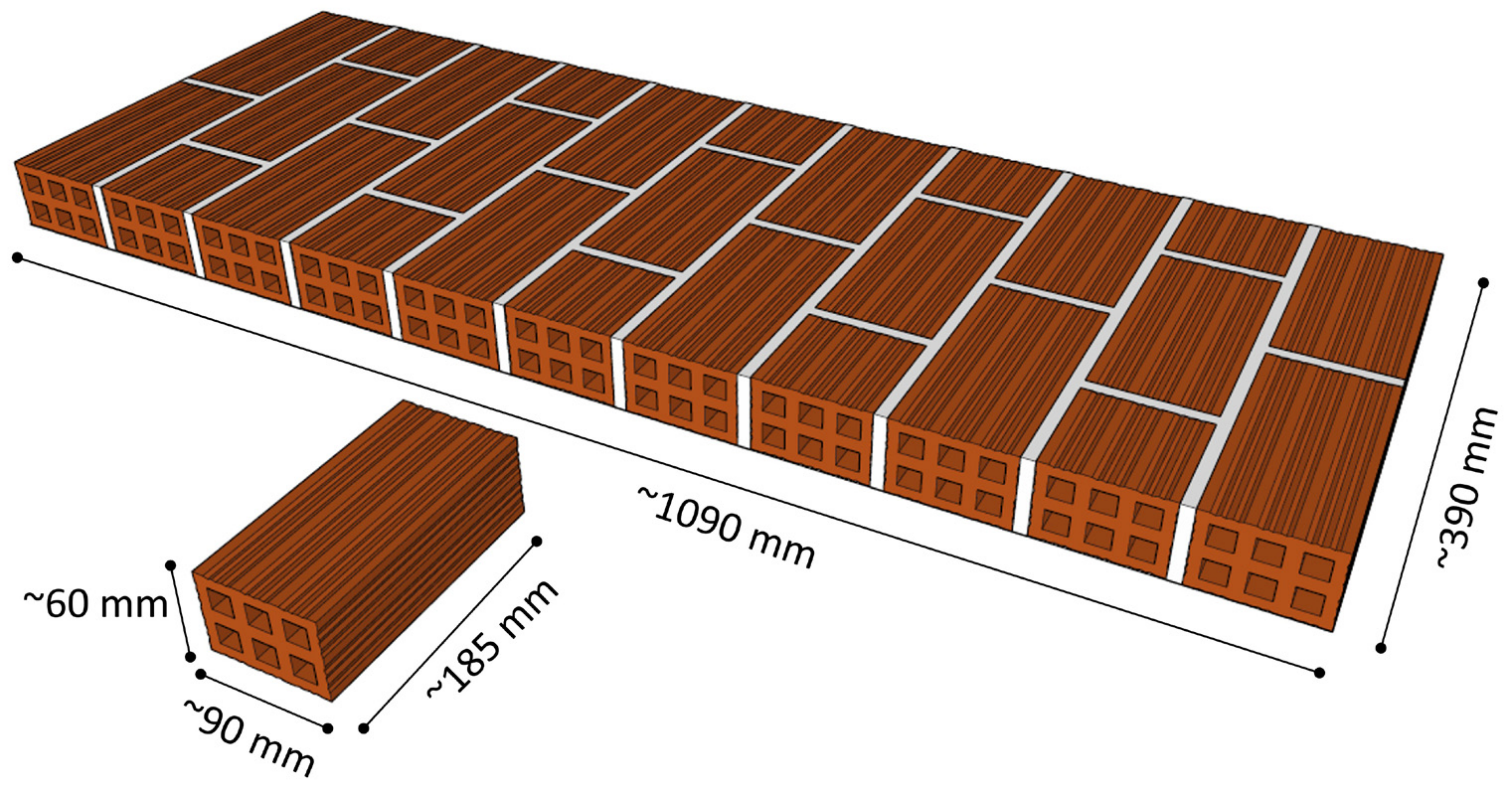
Wall geometry (dimensions in mm).

The single-leaf masonry walls were built using standard fired-clay hollow bricks and cement-lime mortar maintaining overall dimensions of 1090x390x65 mm ( Figure 2). The name of each specimen corresponded to the type of the materials and the layout used ( Table 1). The first part corresponds to the type of the binder (CEM – cementitious, GEO – geopolymer) and the second part has encoded the type of the textile and the layout of the strengthening. For instance, CEM-IG2 is the wall retrofitted using standard cementitious binder (CEM) and a layer of insulation (I), followed by two layers of glass TRM (G2). Likewise, GEO-B1I1 is the wall with geopolymer binder and insulation (I) placed between two layers of basalt textile (B1I1).

**Table 1.** Test specimens and strengthening configurations.

No	ID tag	Insulation	Textile	Binder/matrix material	TRM layers	*ρf* (%)	Layout
1	CEM-G2	NO	Glass	Cement-based mortar	2	0.16	2 TRM
2	CEM-B2	NO	Basalt	Cement-based mortar	2	0.11	2 TRM
3	GEO-G2	NO	Glass	Geopolymer mortar	2	0.16	2 TRM
4	GEO-B2	NO	Basalt	Geopolymer mortar	2	0.11	2 TRM
5	CEM-IG2	YES	Glass	Cement-based mortar	2	0.11	Ins + 2 TRM
6	CEM-IB2	YES	Basalt	Cement-based mortar	2	0.08	Ins + 2 TRM
7	GEO-IG2	YES	Glass	Geopolymer mortar	2	0.11	Ins + 2 TRM
8	GEO-IB2	YES	Basalt	Geopolymer mortar	2	0.08	Ins + 2 TRM
9	CEM-G1I1	YES	Glass	Cement-based mortar	2	0.13	TRM + Ins + TRM
10	CEM-B1I1	YES	Basalt	Cement-based mortar	2	0.09	TRM + Ins + TRM
11	GEO-G1I1	YES	Glass	Geopolymer mortar	2	0.13	TRM + Ins + TRM
12	GEO-B1I1	YES	Basalt	Geopolymer mortar	2	0.09	TRM + Ins + TRM

**Figure 3.**
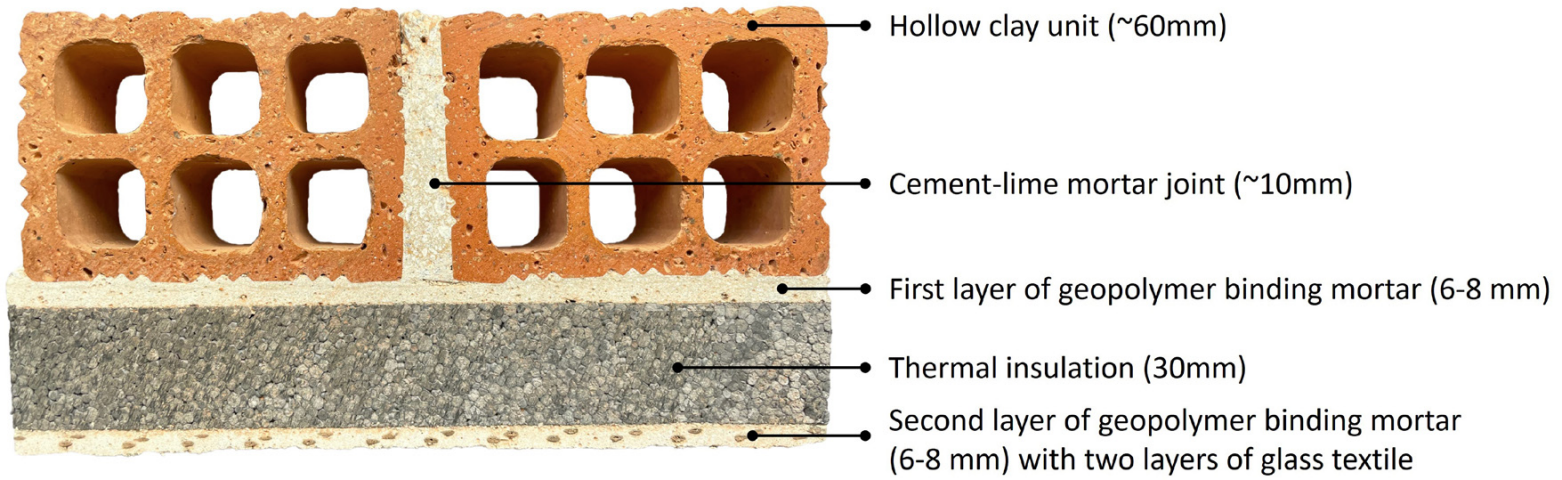
Typical section (part of the longitudinal section through specimen GEO-IG2).

The construction of the walls without insulation followed the same procedure as described in the authors’ study on shear strengthening ( Cholostiakow *et al.*, 2023a). The walls were cleaned from dust and debris and the entire surface of the wall was covered with the first layer of mortar (approx. 3-4 mm). The first layer of textile was then pressed and rubbed into the mortar until it was fully embedded in the binder. Finally, another layer of mortar of about the same thickness was applied to fully cover the textiles and the same procedure was repeated for the second layer. The walls with the thermal insulation, were retrofitted in two steps. In the case of the layout (IG2/IB2), the insulation board was bonded to the masonry surface using the same binder as that used later for the textiles’ matrix. After the binding mortar hardened, two TRM layers were applied to the surface of the insulation. In the case of the layout (G1I1/B1I1), one TRM layer and the insulation board were applied and left for hardening and in the second phase the second layer of TRM was applied on the insulation board’s surface. In both cases, the insulation board after bonding was pressed over its entire surface to ensure that the bond between the board and the masonry is of uniform thickness. Figure 3 shows a typical section through the part of the masonry panel GEO-IG2 retrofitted with two glass TRM layers applied to the surface of the insulation board. 

### Masonry

Standard hollow fired-clay bricks (typically used in Southern Europe) were used to build the walls ( Figure 2). The compressive strength of the brick units was determined according to EN 772-1 ( CEN, 2015) for the direction parallel and perpendicular to the holes and was found equal to 15.5 MPa (COV 16%) and 7.1 MPa (COV 7%), respectively. A conventional cement-lime mortar was used to build the walls using 1:1:5 ratio by volume for CEM II 32.5R cement, lime, and 0/4 mm sand, respectively. The compressive and flexural strength of the mortar were determined on the day of testing according to EN 1015-11 ( CEN, 2019). The measured compressive strength was 9.8 MPa (COV 12%), while the flexural strength was 2.0 MPa (COV 10%).

### Textile reinforcement

**Figure 4.**
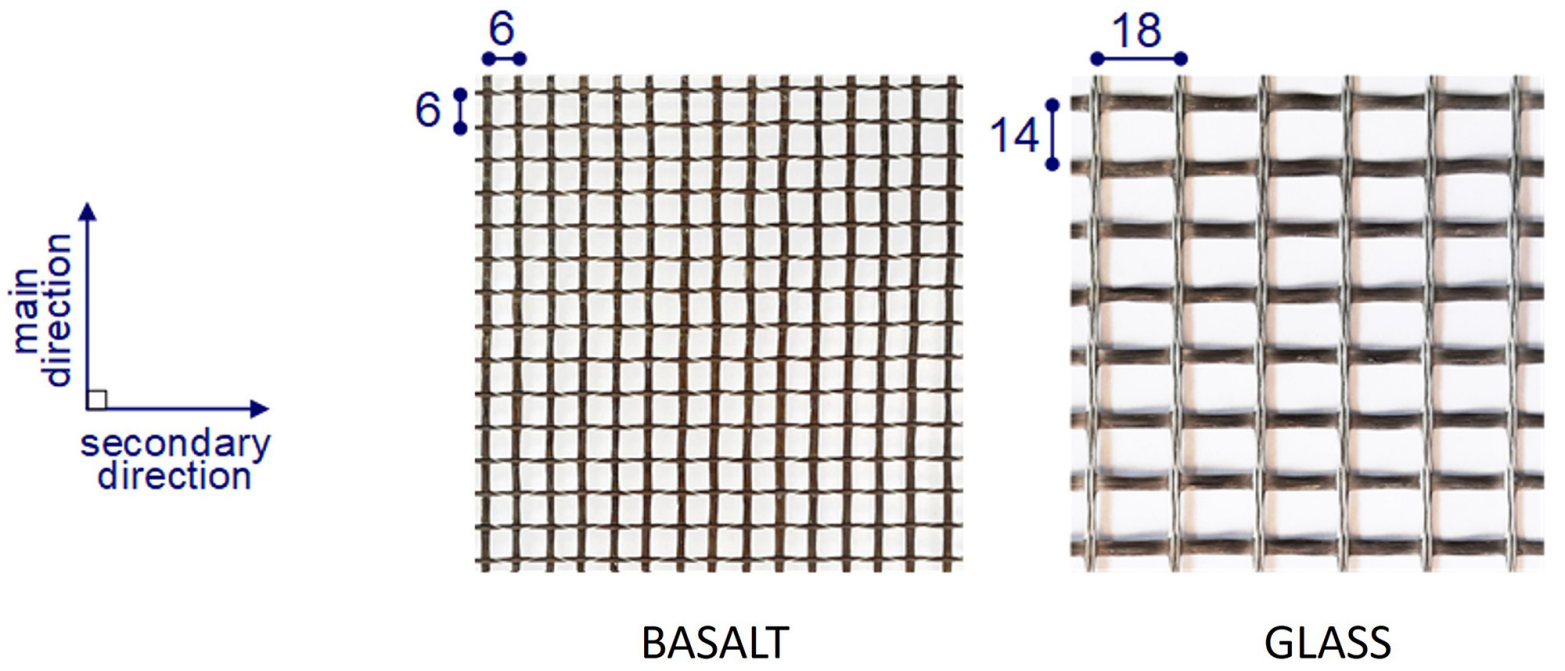
Textile geometry (dimensions in mm).

The study investigated two different types of commercially available textiles: i) 6x6 mm square mesh basalt textile grid; ii) 14x18mm rectangular mesh glass textile grid ( Figure 4). The weight distribution of the fibre rovings was 50-50 % in the case of basalt textile and 51.8-48.2 % for the glass textile. The main (warp) direction of the glass was always oriented along the wall’s large dimension. The basalt textile was epoxy-coated, whereas the glass textile had a styrene-butadiene rubber (SBR) coating. The mechanical properties of both textiles as reported by the manufacturers are listed in Table 2. As can be seen, due to the larger nominal thickness, the glass textiles possessed higher axial stiffness. The reinforcement ratios (see Table 1) for all walls were calculated as *ρ _f_* = *t _f_*/ *d*, where *t _f_* is the total thickness of the reinforcement and *d* is the distance between the outer compression fibre and the centroid of tensile reinforcement ( *d* = 69 mm for walls without insulation, *d* = 87 mm for the walls with 1I1 layout, *d* = 104 mm for the walls with I2 layout).

**Table 2.** Mechanical properties of the textiles.

Mechanical property	Basalt-fibre textile	Glass-fibre textile
Elastic modulus - dry fibres (GPa)	89	82
Total weight (g/m ^2^)	250	360
Weight without coating (g/m ^2^)	214	280
Nominal thickness (main dir., mm)	0.039	0.056
Axial stiffness - dry fibres (main dir., kN/mm)	3.47	4.48
Tensile capacity (main dir., kN/m)	60	77

### Matrix/binding mortars

A commercially available one-component cement-based mortar was selected as a fair benchmark for the designed geopolymer mortar. The mortar dry mix comprised cement, aggregates with maximum size of 1.3 mm and polypropylene microfibers. The desired workability was achieved by mixing water at fixed water-to-dry-mix ratio of 0.23 (by weight).

The geopolymer mortar was developed at the University of Thessaly's Laboratory of Concrete Technology and Reinforced Concrete Structures. The mortar consisted of a high reactivity metakaolin as a precursor with particle size *d _95_* = 80 μm and 95% aluminosilicate content, which derived from the calcination of kaolinitic clay. As the alkaline activator, a potassium silicate solution with a weight ratio of SiO _2_/K _2_O of 1.07 was used. Limestone sand with a maximum particle size of 1 mm was used as filler. Polypropylene fibres of 6 mm length were also added with a volume fraction of 1% to enhance the flexural strength and reduce the onset of cracking and shrinkage.

**Figure 5.**
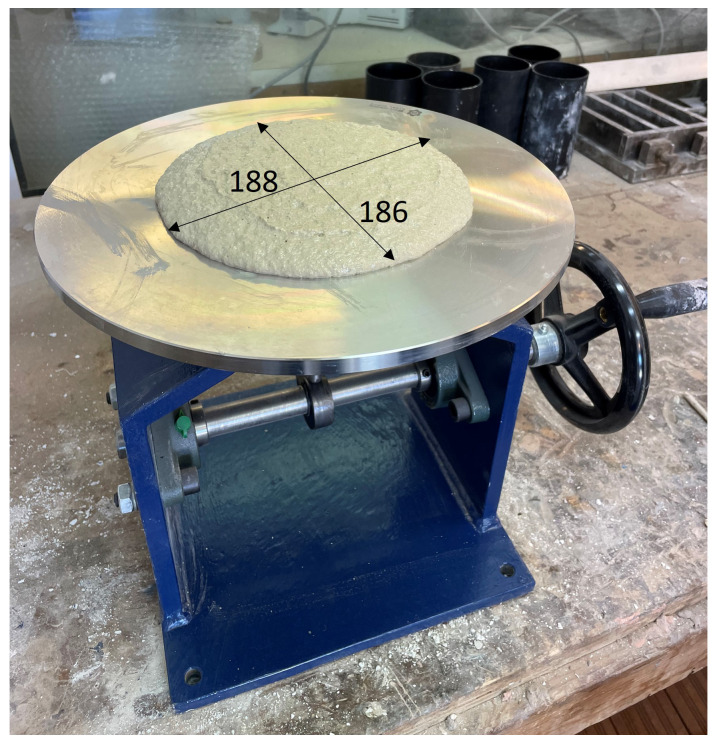
Fresh mortar mix consistency test for geopolymer mortar (measurements in mm).

The properties of both matrix mortars were investigated, and the results are presented in Table 3. The consistency of the fresh mortars was determined experimentally ( Figure 5) according to EN 1015-3 ( CEN, 1999). The recorded flow values of the cement-based mortar were equal to 180 mm and 184 mm measured at right angles to one another. The same property was also recorded for the geopolymer mortar which had a flow equal to 186 mm and 188mm, respectively. The tests on hardened cement-based mortar samples carried out according to EN 1015-11 ( CEN, 2019) after a period of 28 days, reveal flexural strength and compressive strength equal to 4.9 MPa and 22.2 MPa, respectively. Similarly, the tests on hardened geopolymer mortar samples reveal flexural strength and compressive strength equal to 5.5 MPa and 44.3 MPa, respectively.

**Table 3.** Properties of the binders.

Binder/matrix material	Average flow value (mm)	Flexural strength (MPa)	Compressive strength (MPa)
CEM	182	4.9 (4%)	22.2 (7%)
GEO	187	5.5 (18%)	44.3 (9%)

### Thermal insulation

Standard 30 mm-thick expanded graphite polystyrene insulation (EPS) boards with thermal conductivity value of 0.03W/mK were employed as insulation materials for the energy retrofitting of the walls. According to the manufacturer, the guaranteed compressive strength and the shear modulus of the board were equal to 0.02 MPa and 1.0 MPa accordingly. Figure 3 shows a section through the masonry panel GEO-IG2 retrofitted with two glass TRM layers applied to the surface of the insulation board. As can be seen, the developed geopolymer mortar was able to fill the contact area between the wall and EPS board as well as provided uniform embedment for the textile reinforcing mesh.

### Test setup

**Figure 6.**
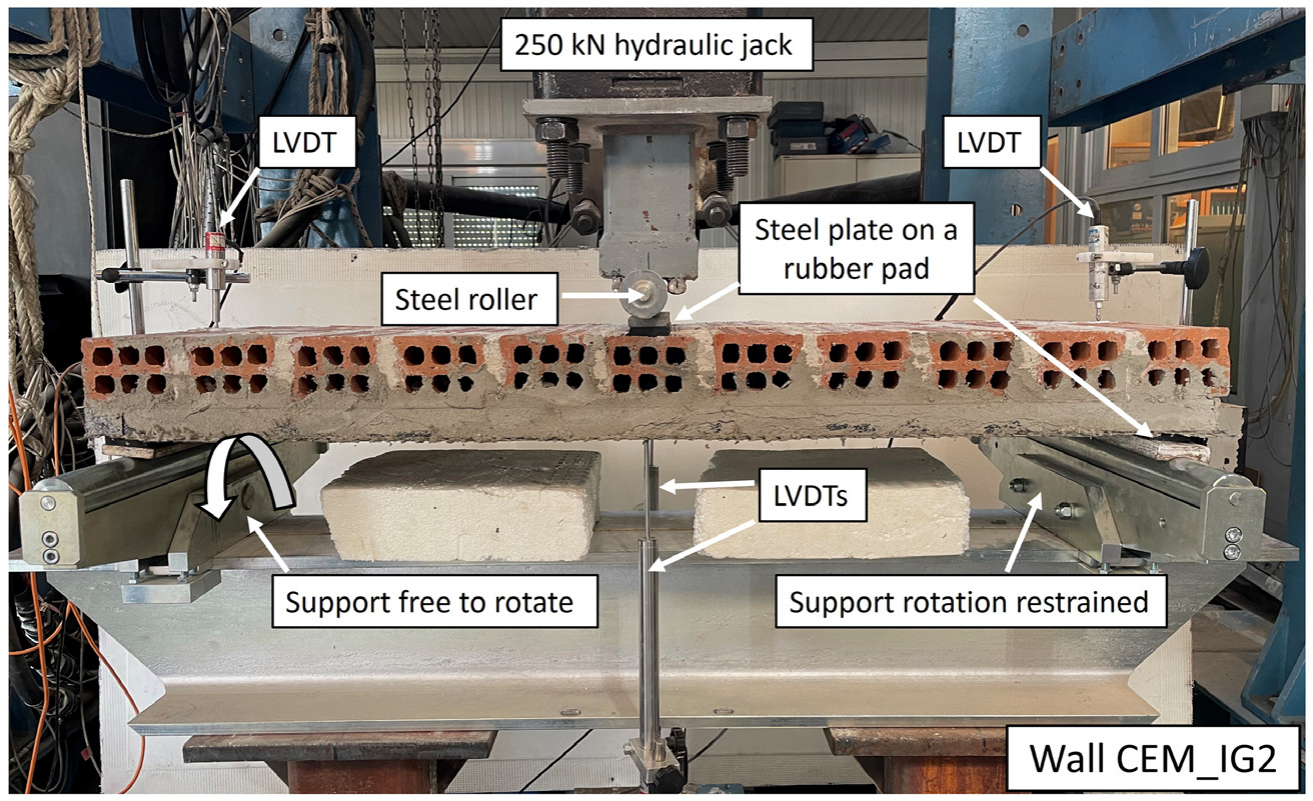
Test setup and instrumentation.

The walls were tested as simply supported over a 1000 mm clear span with the load being applied at midspan ( Figure 6). The load was applied and transferred to the supports across the entire walls’ breadth, i.e. 390 mm. To spread the load and avoid any local masonry damage in the vicinity of the loading points, 50x390x10 mm steel loading and bearing plates were used together with 10 mm-thick rubber pads. One of the supports was free to rotate, thus helping to accommodate any eventual imperfections in the walls’ plane. The load was applied at a displacement rate of 0.04 mm/s using a 250 kN servo-hydraulic actuator. The midspan deflection was measured using two linear variable differential transformers (LVDT) placed in the centre. The settlement of the wall due to the deformations in the thermal insulation was captured using two additional LVDTs installed on the wall above the supports. The net deflection was calculated as the average midspan deflection minus the wall’s average settlement measured at the supports. The data was recorded by a fully automated data acquisition system at a sample rate of 2 Hz.

## Results

### Failure patterns and cracking behaviour

**Figure 7.**
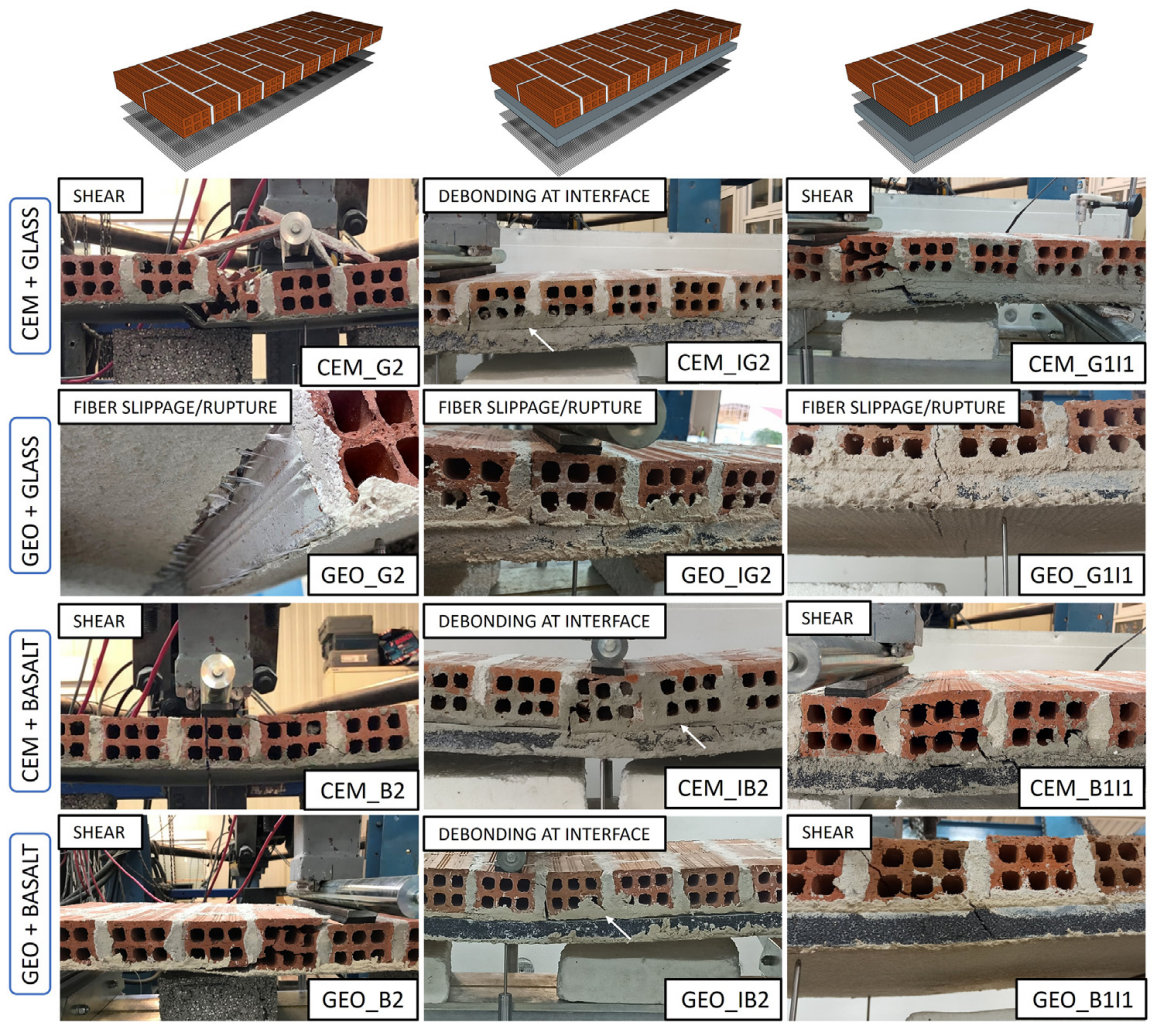
Failure modes for all tested walls.

The failure patterns for all walls are shown in Figure 7. The addition of two TRM layers can substantially increase the flexural capacity of the walls beyond their shear capacity. Hence, it was expected that the failure mode will change from flexural to shear. The reference walls with two TRM layers and CEM binder (without insulation), CEM_G2 and CEM_B2, showed typical diagonal shear failure, indicating that both TRM layers were mobilized and efficiently resisted tension. The type of the textile did not have any significant influence on the failure, which suggests that both textiles were well mobilized in the CEM binder. However, a similar agreement was not seen when GEO binder was used. An analogous structural response and failure was achieved only by GEO_B2, which exhibited identical failure mode as its CEM counterpart, i.e. diagonal shear crack, thus indicating good ability to bridge the cracks in the binder and redistribute the load over the TRM overlay. On the other hand, the glass textile in GEO_G2 did not exhibit a full composite action with the GEO binder but a more local and premature type of failure; the glass textile rovings ruptured in the vicinity of the maximum bending moment showing also signs of partial slippage within the GEO matrix.

Specimens CEM_IG2, GEO_IG2, CEM_IB2 and GEO_IB2, retrofitted with a thermal insulation board followed by two TRM layers did not experience any diagonal shear cracking. After the initial cracking (in the TRM and then in the masonry), the walls with CEM binder developed a vertical crack along the mortar joint-brick interface leaving the insulation board undamaged. With the increasing load and further opening of the cracks in the masonry, a horizontal crack propagated at the board-masonry interface (see white arrows in Figure 7) towards the support, in consequence, leading to debonding of the insulation board from the masonry substrate and separation of the masonry panel into two pieces. The GEO-IB2 wall exhibited similar behaviour at the ultimate condition with the exception that no major cracks were observed in the TRM overlay. Debonding failure was not seen in the GEO_IG2 wall, which failed in the same manner as the reference wall GEO_G2, which was due to textile rupture/slippage again, indicating that this specific combination of materials is not suitable for this kind of application.

The walls with the “sandwich” layout, having EPS insulation board bonded between two TRM layers (1I1 layout), showed failure similar to the walls with REF layout, i.e. diagonal shear crack through the wall and also through the insulation board. Both the CEM_G1I1 and CEM_B1I1 walls failed in the same manner as their reference walls without the insulation boards, showing excellent bond and full composite action up to failure. As such, evidencing that such a strengthening configuration leads to better structural response and offers better integration for the TRM and the EPS board. Similar to the performance in the previous tests, when the GEO binder was combined with the glass textile (GEO-G1I1 wall) the failure was premature; the wall failed prematurely in flexure before reaching shear capacity of the masonry panel. In contrast, when the GEO binder was combined with the basalt textile (GEO_B1I1 wall) the specimen showed excellent composite action and outperformed its cementitious counterpart (CEM_B1I1).

The experimental observations on the cracking behaviour and failure mechanisms evidence that both the strengthening layout and the type of the binding mortar can alter the failure modes of masonry walls subject to out-of-plane loading. The I2 type of layout appears to change the failure pattern from diagonal shear to debonding of the insulation board, hence leading to poor integration of the thermal retrofitting with the TRM. The 1I1 layout leads to better composite action between the masonry, thermal insulation and TRM retrofitting layers. The use of the GEO binder together with the glass textile led to the worst performance and failure mode of the walls at the onset of cracking in the TRM overlay. The next sections discuss in detail the effects of the variable parameters on the structural response of the walls.

### Effect of the retrofitting layout

A composite overlay combining a standard TRM system with thermal insulation boards can improve both energy efficiency and structural capacity of the masonry walls. Provided that full-composite action between the materials is maintained, the integration of the EPS insulation board into the system can also lead to additional benefits in the structural out-of-plane response. Assuming that deformations across the wall’s section are not far from linear, the application of a 30 mm-thick EPS insulation board should increase the lever arm between the compressive force in the masonry and the tensile force in the textile reinforcement, thus contributing to the structural performance and making this system more effective. The three TRM layouts examined in this paper varied in the height of the lever arm; the walls with the standard TRM application directly to the surface of the wall had the shortest lever arm, whereas the walls with two TRM layers applied on the 30 mm EPS insulation board had the longest lever arm of internal forces. The “sandwich” layout having the EPS board between TRM layers represented a lever arm length between the two. As such, if the deformations are linear or approximately across the layers of the composite the post-cracking bending stiffness and the overall performance of the walls with integrated energy retrofitting should be substantially higher compared to the walls with standard TRM application.

**Figure 8.**
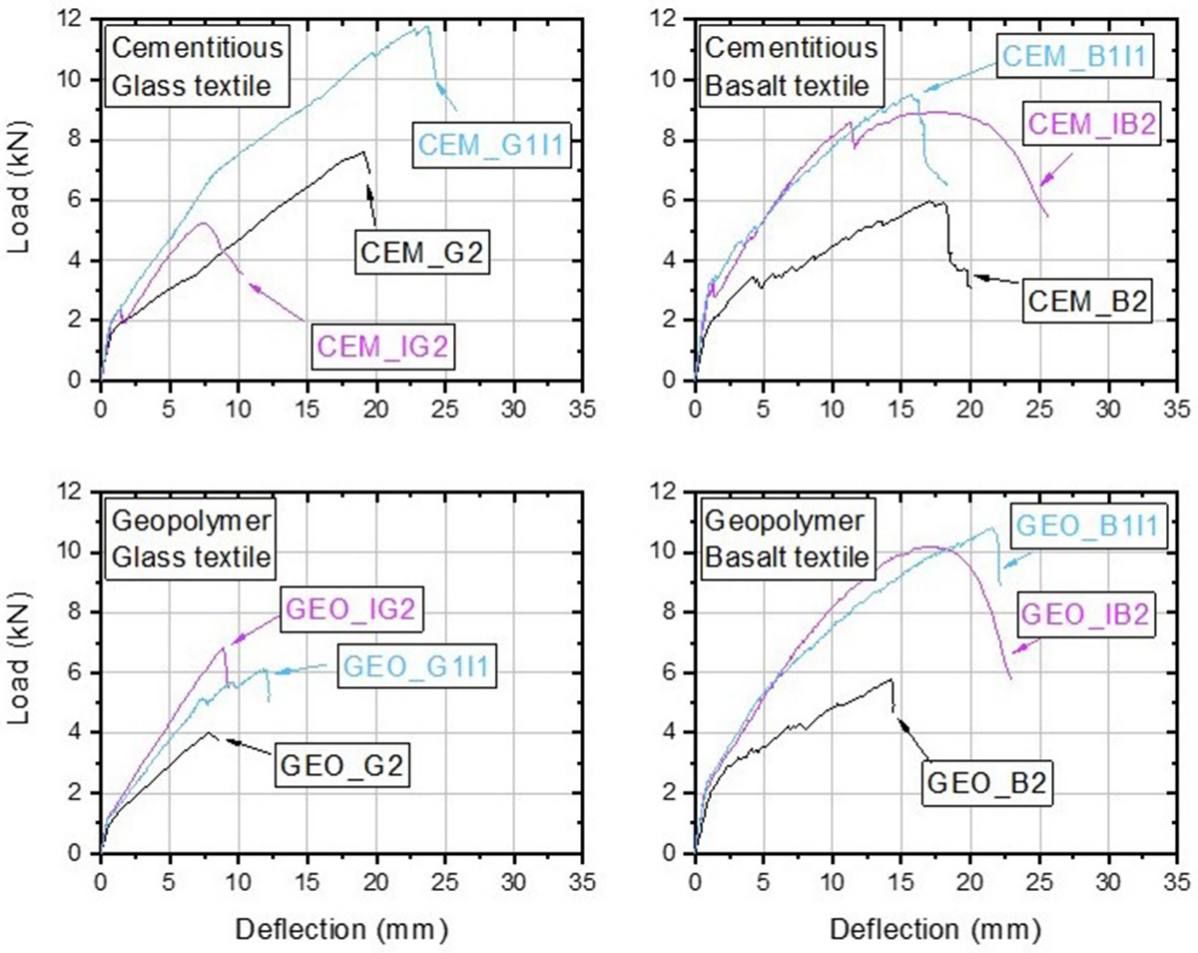
Load-deflection curves: effect of the retrofitting layout.

The load-deflection curves for all tested walls are shown in Figure 8. Each plot shows a comparison of three walls with a different strengthening configuration while the remaining parameters such as type of binder and mortar are kept constant, hence, allowing to see how each type of retrofitting layout affected the overall structural response. The main test results are also summarized in Table 4, where the benefits and the effectiveness of the integration of structural strengthening with energy retrofitting are assessed in terms of gains in post-cracking flexural stiffness and maximum bending capacity ( Cholostiakow *et al.,* 2023c). The experimental post-cracking bending stiffness was estimated between the onset of cracking and the peak load. For this part of the graph a linear fit was used to estimate the slope (stiffness) after the initial cracking.

**Table 4.** Main test results.

No	ID tag	Load at failure (kN)	Flexural stiffness after cracking (kN/mm)	Average midspan deflection measured at peak load (mm)	Failure mode
1	CEM_G2	7.62	0.34	19.1	Shear
2	CEM_B2	5.97	0.22	16.9	Shear
3	GEO_G2	4.04	0.41	7.8	Fiber slippage/rupture
4	GEO_B2	5.79	0.25	14.2	Shear
5	CEM_IG2	5.25 (-31.1)	0.62 (82.4)	7.5 (-60.7)	EPS board debonding
6	CEM_IB2	8.94 (49.7)	0.60 (172.7)	18.2 (7.7)	EPS board debonding
7	GEO_IG2	6.82 (68.8)	0.67 (63.4)	8.9 (14.1)	Fiber slippage/rupture
8	GEO_IB2	10.2 (76.1)	0.52 (108.0)	17.3 (21.8)	EPS board debonding
9	CEM_G1I1	11.76 (54.3)	0.44 (29.4)	23.7 (24.1)	Shear
10	CEM_B1I1	9.52 (59.5)	0.43 (95.5)	15.8 (-6.5)	Shear
11	GEO_G1I1	6.0 (48.5)	0.49 (19.5)	11.8 (51.3)	Fiber slippage/rupture
12	GEO_B1I1	10.8 (86.5)	0.41 (64.0)	21.6 (52.1)	Shear

*Values of relative percentage increase (or decrease) compared to the reference walls are given in the parentheses.

It should be noted that such slender walls without any retrofitting would fail at very small load or even under their own weight ( Papanicolaou *et al.*, 2011). Hence, bare URM walls were not examined experimentally, and any OOP capacity offered by URM was deemed as negligible. In this context, the walls without insulation served as reference specimens, and hence relative comparisons were made to the TRM-retrofitted walls without insulation (REF), thus revealing the overall efficiency of the holistic retrofitting approach (structural plus energy retrofitting). The strengthening efficiency was calculated as the capacity increase between a specimen retrofitted with the combined approach and a specimen with standard TRM structural retrofitting.

Owing to the relatively high mechanical properties of textiles and excellent compatibility of binding matrix with the masonry substrate, two TRM layers substantially increased the OOP capacity of the walls and served as a viable and effective structural retrofitting system. The largest peak load was attained by CEM_G2 and was equal to 7.68 kN, whereas the lowest peak load was recorded by GEO_G2, which failed at a load of 4.15 kN. 

**Figure 9.**
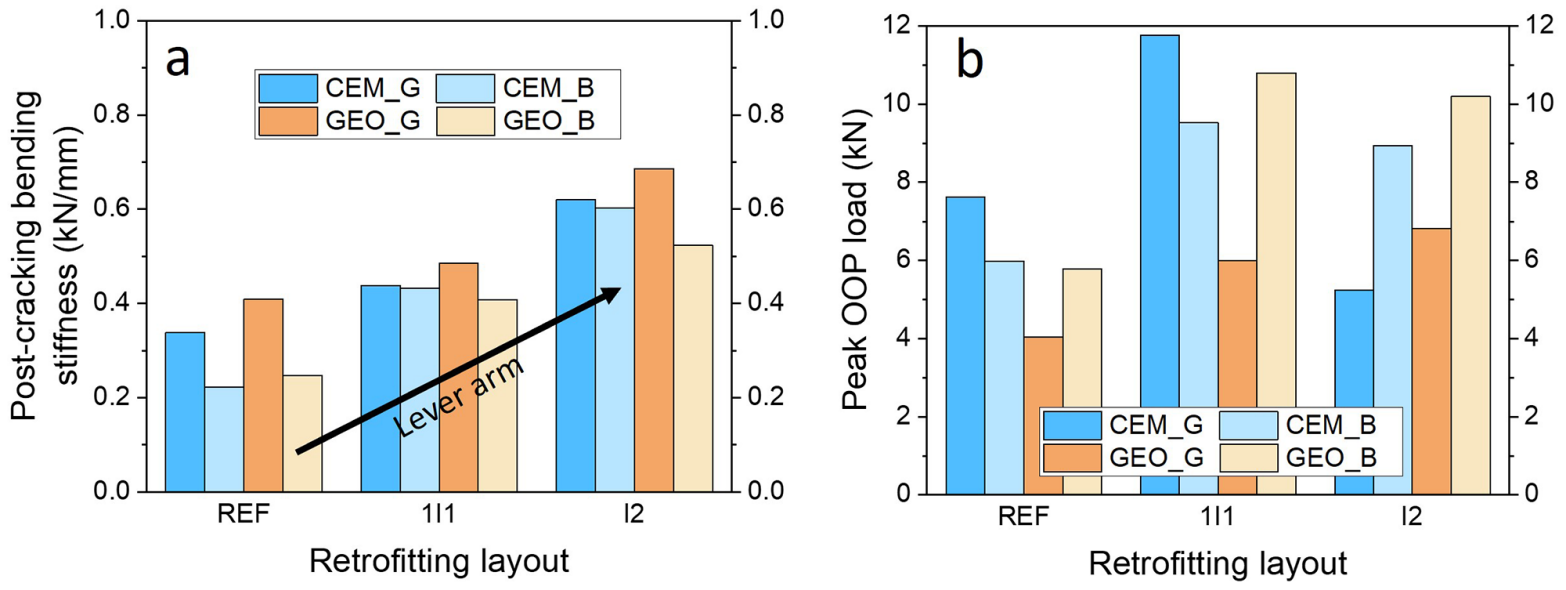
Contribution of the retrofitting layout to **a**) experimental post-cracking bending stiffness; **b**) experimental OOP capacity.

The walls combining structural and energy retrofitting clearly exhibited structural performance superior to the REF walls, both in terms of bending stiffness and load capacity ( Figure 9). The post-cracking stiffness increased with the increasing height of the lever arm, showing an approximately linear trend. Compared to the REF walls, the increase ranged from 29% to 95% for the 1I1 configuration, whereas for I2 configuration the recorded stiffness increase was between 63% and 108%, depending on the materials used. The approximately proportional increase across the examined retrofitting layouts evidence that both sections incorporating thermal insulations were effectively engaged after the first cracking, maintaining the composite action between the TRM, EPS board and the masonry wall. It also appears that the addition of weak materials such as an EPS board does not affect the stress distribution in bending and an approximately linear distribution can be assumed for calculations. In turn, the load resisted by the TRM was effectively transferred to the wall and enabled achieving a high load-capacity increase. However, as discussed before, the type of retrofitting layout led to a different failure behaviour at the ultimate condition, and thus influenced the ultimate load capacity. All walls with energy retrofitting apart from CEM_IG2 exhibited larger OOP capacity compared to the REF walls. Although the largest stiffness was expected and then indeed recorded for the I2 layout, these walls failed earlier than their 1I1 counterparts due to the different failure behaviour (see Figure 7). The largest increase with respect to the REF walls was recorded for GEO_B1I1 and was equal to about 86%, owing to the excellent bond to the masonry substrate, which enabled effective composite action between the materials until reaching shear capacity of the masonry. The walls retrofitted with I2 configuration in general showed decent increase in the OOP capacity up to 76% (GEO_IB2), with the exception of CEM_IG2 which failed at a very low load. However, the post-failure inspection of the wall revealed imperfect mortar application between the EPS board and the masonry with several air gaps between them. These gaps can reduce the bond strength between the two materials, thus leading to lower OOP load-capacities. Hence, it should be highlighted that in practice such a layout can be risky as direct application of an EPS board on the wall requires a relatively even wall surface, which is often not the case for substandard masonry structures. Analysing Figure 8 it can be clearly seen that the combination of geopolymer and glass textiles led to the poorest performance, showing strong mortar effect on the test results and this will be discussed in the next sections.

### Performance of the geopolymer mortar

**Figure 10.**
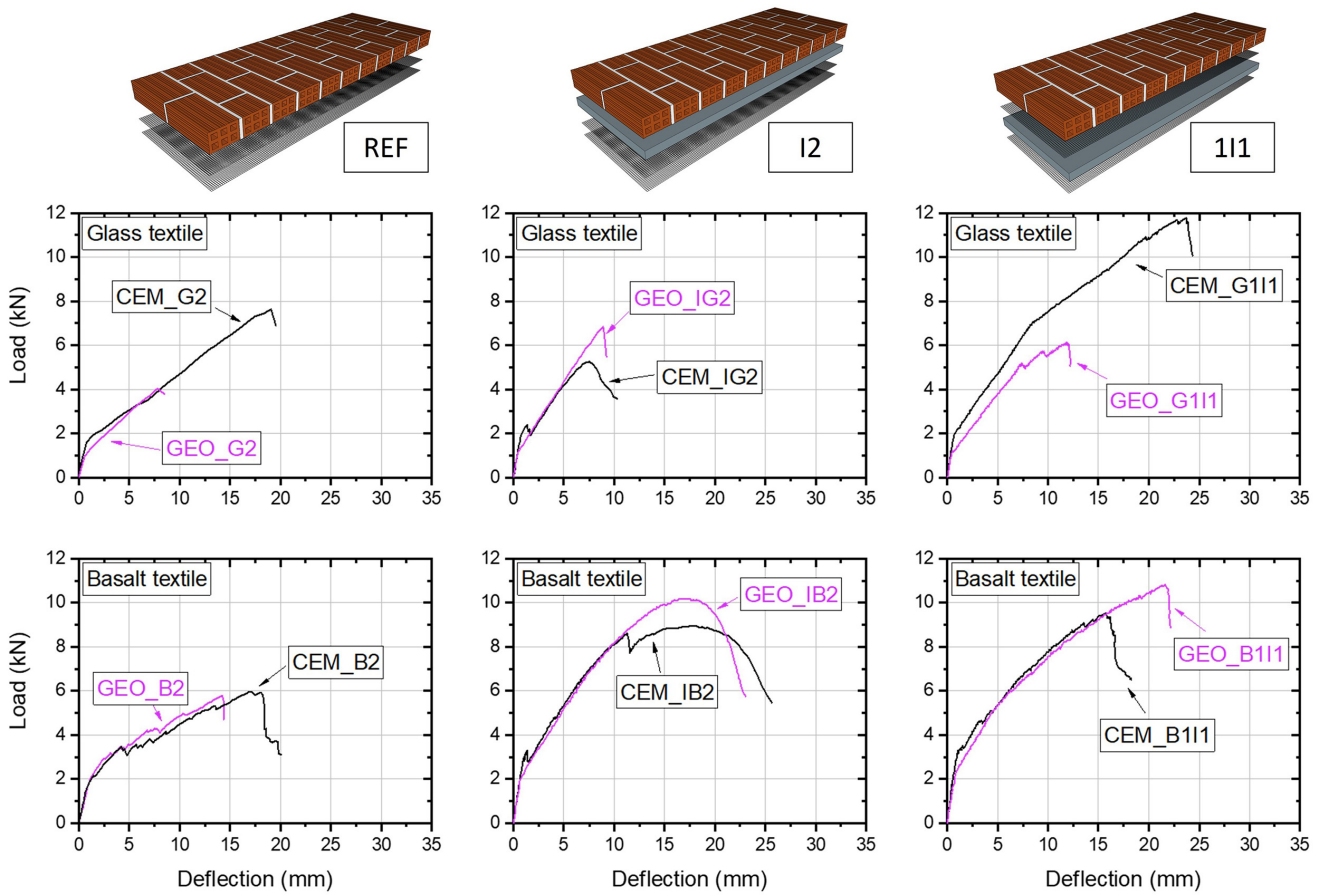
Load-deflection curves: effect of geopolymer mortar on structural behaviour of the walls.

The effect of the mortar (binder) type on the structural OOP performance of the masonry walls is illustrated in Figure 10. Each pair of curves comprise the structural responses of two walls varying only in the type of binder used as a matrix for the textiles (CEM or GEO). As such, the effect of the geopolymer mortar on the structural response can be directly assessed and compared to the standard cement-based binder. The REF walls retrofitted with two layers of glass textiles without thermal insulation, CEM_G2 and GEO_G2, though having slightly different cracking strength, showed almost identical load path after the first cracking. Nevertheless, GEO_G2 did not manage to match the capacity of its cementitious counterpart and failed in a flexural manner, prior to reaching shear capacity of the masonry. The result was that GEO_G2 failed due to early rupture of the glass textile rovings in the vicinity of the flexural cracks (see also Figure 7) attaining only about half of the OOP capacity of CEM_G2. On the other hand, the walls with two layers of the basalt textile, CEM_B2 and GEO_B2, showed similar load response through the entire loading history and produced the same failure mode. These results provided the first indication that - if combined with a suitable textile - geopolymer mortar can lead to similar performance compared to standard cement-based mortar. It is important to highlight that for the same textile there might be different results if different mortars are used; this is mainly attributed to incompatibility issues between geopolymer and specific types of textiles coating materials. The latter is being discussed later.

GEO_IG2 and CEM_IG2 walls retrofitted with a layer of thermal insulation followed by two glass TRM layers showed comparable load displacement paths, but earlier cracking was observed in the GEO wall. The cracking in CEM_IG2 occurred at a higher load was accompanied by a visible load drop and change in bending stiffness which is characteristic to brittle materials. GEO_IG2 failed reaching approximately the same deflection as GEO_G2 and through the same failure mechanism (fibre slippage/early rupture), thus confirming that geopolymer and glass textile did not produce a desired composite action. Likewise, for the B2 series similar responses were achieved for the pair GEO_IB2 and CEM_IB2, with the former showing better performance and load capacity about 23% higher than the wall retrofitted with CEM-based binder. Whilst load drops and stiffness change associated with initial cracking and intermediate crack debonding can be easily identified for CEM_IB2, the response of GEO_IB2 was again non-linear throughout the entire loading history, indicating good compatibility of the GEO matrix with basalt textiles and less susceptibility to cracking.

A strong influence of the mortar type was also seen between walls CEM_G1I1 and GEO_G1I1 having thermal insulation between two glass TRM layers. CEM_G1I1 developed the largest load bearing capacity equal to 11.76 kN and produced significant cracking in the TRM overlay. Again, the wall with GEO binder was not able to outperform its counterpart wall with CEM binder and the wall failed at 6 kN due to early rupture of the textiles, shortly after the development of a critical flexural crack in the TRM overlay at midspan ( Figure 7). CEM_B1I1 and GEO_B1I1 performed significantly better and showed similar behaviour irrespectively to the type of binding mortar used with the GEO wall developing about 12% larger load capacity.

In general, the walls with cementitious binder exhibited clear initial cracking, whereas in GEO-retrofitted walls no clear first cracking point was recorded during the test. Instead, the change in stiffness was more gradual and was represented by a non-linear behaviour until reaching the ultimate failure. Although the initial stiffness was similar for GEO and CEM walls, the initial cracking occurred at a lower load in the walls with GEO binder and this was more pronounced when glass textiles were used. This evidence that this type of GEO mortar had a substantial influence on the performance of glass textiles already at the early stages of loading. The basalt textiles performed much better in both types of mortars showing the best overall performance, and this is discussed in detail in the next section.

### Performance of the textile reinforcement

**Figure 11.**
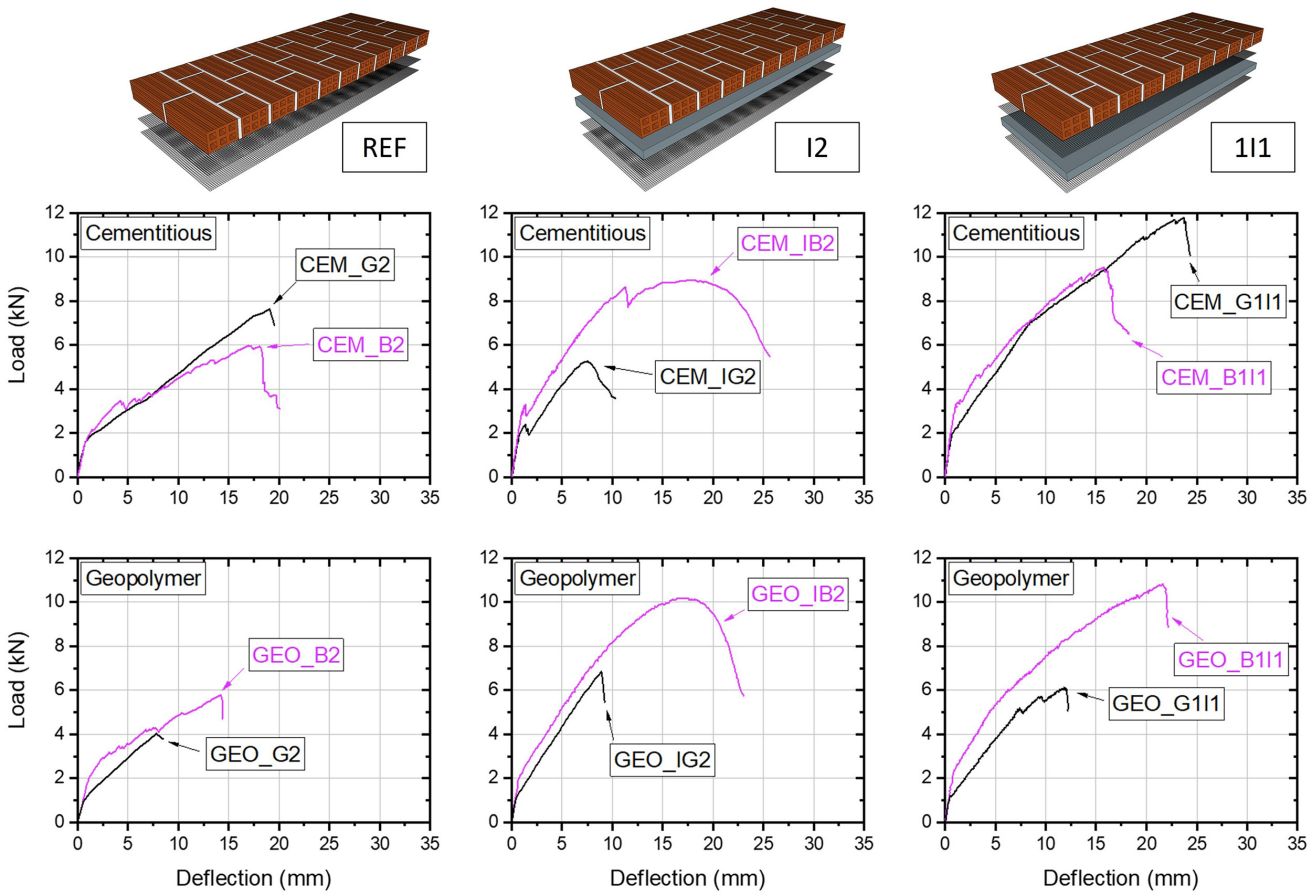
Load-deflection curves: effect of textile reinforcement on structural behaviour of the walls.

The effect of textile reinforcement on the structural response of the walls is shown in Figure 11. The type of the textile influenced the structural response of the walls, and this effect was related to i) the mechanical and geometric properties of the two textiles, and ii) the chemical compatibility with the binder. In general, walls with cement-based binder exhibited earlier cracking but slightly stiffer post-cracking response when glass textiles were used. For instance, CEM_B2 and CEM_B1I1 with basalt textiles exhibited softer post-cracking response and lower ultimate capacity than their counterparts reinforced with glass textiles. This was not the case only for CEM_IG2 but this was caused by the workmanship and the authors believe that a similar trend would have been achieved if the bond between the insulation board and the masonry was improved (see also the discussion in the previous section). An enhanced performance of glass textiles was expected and can be attributed to the fact that the glass textile had a larger thickness (0.056mm) than the basalt textile (0.039mm), thus resulting in slightly larger axial stiffness ( Table 2). Therefore, the difference between the two textiles combined with CEM binder was rather minor and was merely related to the textile’s mechanical properties.

When the geopolymer binder was used the effect of the textile type appeared to be more significant. As it was reported in the previous sections, this specific type of glass textile reinforcement showed poor performance in the geopolymer binder and ruptured at relatively low load levels, not contributing much to the OOP capacity of the walls. The glass textiles in GEO_G2 ruptured at a load corresponding to about 70% of the capacity of GEO_B2 – its counterpart wall with basalt textiles which failed in shear. Similar results were seen for GEO_IG2 and GEO_G1I1, which failed at comparable loads, not contributing much to the load capacity nor the integrity of the entire composite system. On the other hand, the use of basalt textiles improved the cracking load and allowed for larger utilisation of the textile reinforcement. Compared to the REF walls, GEO_IB2 and GEO_B1I1 developed an increase in the OOP capacity of about 60% and 86%, respectively, showing good compatibility not only with the geopolymer binder but also with the EPS board. Both retrofitted showed capability to achieve composite action till reaching the load of 10 kN, thus suggesting that the specific glass textiles used in this study are not suitable reinforcing materials for this type of binder and application. This is probably attributed to the chemical incompatibility between the textile’s coating material (SBR coating) and the geopolymer binder developed at the lab.

## Discussion

The results of this experimental study clearly indicate that geopolymers can be used as binders for textiles in standard TRM applications and are able to produce similar or even improved performance compared to standard cement-based binders. However, OOP test results on masonry walls showed that certain combinations of materials can lead to poor structural performance; the investigated glass textiles with SBR coating exhibited poor bond conditions with the geopolymer binder, thus leading to premature failure. On the other hand, glass textiles showed good performance in CEM binders, with the largest OOP capacity developed by CEM_G1I1.

It is worth noting that identical materials were already tested by the authors in an earlier experimental study examining shear behaviour of masonry walls retrofitted with TRM ( Cholostiakow *et al.*, 2023a) and quite contrasting observations were made regarding glass textiles in a geopolymer matrix. In diagonal compression tests on TRM-retrofitted walls, the glass textiles embedded in the same GEO matrix led to the performance levels surpassing elements retrofitted using CEM binder. Moreover, the GEO matrix proved to be more deformable and able to meet the same shear strength levels at larger shear strain regardless of the textiles used. In the flexural retrofitting discussed within the scope of this paper, the deformability of the walls seems to be limited for a composite formed by glass textiles and a GEO matrix, and all three walls with this combination failed at the onset of cracking in the TRM, before reaching the capacity of masonry. Whilst this could be a bond problem or the incompatibility of the two materials (geopolymer and glass textile) it is uncommon that such different behaviours were achieved in shear and bending. Therefore, this issue warrants further investigation to determine the origins of the different behaviour in flexural and shear applications, and thus provide more detailed recommendations on the use of textiles and geopolymer binders.

The attempts at the integration of TRM and standard thermal insulation systems into one holistic retrofitting approach were successful and led to many useful insights into the structural performance of different retrofitting layouts. The tests confirmed that additional OOP capacity can be expected when thermal insulation is included, and this is because of the increase in the lever arm of internal forces. This has been observed in the past studies and similar conclusions have also been reported (e.g. Triantafillou *et al.*, 2017). Even though IG2 seemed to be the most effective configuration due to the largest lever arm, such a layout caused incompatibilities between the masonry and EPS board, eventually leading to an undesired failure mode like debonding. Similar observations were reported elsewhere ( Karlos *et al.*, 2020), which conclude that a combined TRM and insulation out-of-plane retrofitting scheme is effective only when proper connection between the different layers is maintained. The debonding of the EPS board can be associated with intermediate crack debonding phenomena occurring due to the opening of intermediate cracks in the masonry and the transfer of tensile stresses to the EPS board, causing high interfacial stresses at the wall-board interface. Another limitation of such a configuration is the lack of shear resisting capabilities in the case when the retrofitted wall also requires shear retrofitting, e.g. retrofitting of masonry infills. Hence, for practical applications (e.g. in masonry infills) the approach utilizing the 1I1 layout is much more appropriate and efficient.

The integration with thermal insulation has also a potential to improve the OOP deformation capacity at the peak load, which is very desirable when buildings are subjected to large deformation demands, for instance during strong ground motions. For the CEM binder the best deformation performance was achieved by CEM_G1I1, which exhibited deformation at peak capacity about 24% larger than the REF wall without thermal insulation integrated into the retrofitting system. On the other hand, the remaining walls with CEM did not show any significant improvement in the displacement capacity when EPS boards were introduced. The deformation capacity was better in the walls with GEO binder and basalt textiles, which showed displacement at peak about 50% larger than the REF counterpart specimens regardless of the retrofitting layout, and this is in a good agreement with another study on integrated energy retrofitting ( Gkournelos *et al.*, 2020).

The tests carried out within the scope of this paper clearly showed that the integrated “sandwich” type of application enables to achieve large utilisation of the fibres within the matrix, increases the OOP load capacity and helps to accommodate larger displacement demands than the standard TRM retrofitting without insulation. The effective combination of low carbon footprint materials like a metakaolin-based geopolymer matrix, cost-effective basalt fibres and EPS insulation boards enables achieving a great balance between sustainability, cost, energy performance and structural efficiency, thus contributing towards the development of a new generation of resilient materials for construction.

## Conclusions

Based on the experimental tests and the discussion the following conclusions can be drawn:

- The integration of the two systems led to additional structural benefits; due to the increase in the lever arm, the walls with insulation board exhibited increase in the post-cracking bending stiffness and ultimate OOP capacity. In other words, the integrated system is even more effective than a standard TRM application. The post-cracking bending stiffness increased approximately proportional to the lever arm, suggesting that stress distribution in the materials is not far from linear. The OOP capacity depended on the failure behaviour and the largest increase in the peak load was recorded for the schemes with the “sandwich” (1I1) type of layout.
- The walls with integrated EPS board in 1I1 layout and the walls without insulation exhibited the same failure mode (diagonal cracking), thus confirming that the integration of the two systems was successful. The layout I2, showed a different failure mechanism associated with debonding of the EPS board from the masonry and disintegration of the system, thus appearing as a less preferable retrofitting scheme.
- Geopolymers were able to replicate the behaviour of their cement-based counterparts; however, only for certain combination of the materials. The composite formed of this specific geopolymer and glass textile showed poor performance and immediate failure at the onset of cracking. Hence, this combination of materials is not suitable for flexural retrofitting; however, it can still be used for shear strengthening applications as reported in another authors’ study on masonry infill walls ( Cholostiakow *et al.*, 2023a). More research on bond behaviour of textile reinforced geopolymer mortars is due to reveal which combination of materials leads to the best performance both in flexure and shear.
- Even though glass textiles possessed slightly larger axial stiffness than basalt textiles, both performed well in the cementitious binders. The glass textiles, however, did not engage in the geopolymer binder. More research is warranted to determine whether it is because of the type of coating or the chemical bond between the two materials.

Based on this paper as well as the past research studies it is clear that TRM and EPS can be well integrated into one robust composite system. The combination of geopolymer binder, basalt textiles and EPS boards is capable to create a system, which can largely improve shear, flexural and energy performance of existing buildings utilizing smart, sustainable and more environment-friendly materials. Given that, the next research should focus on retrofitting large scale elements, like masonry infilled RC frames, subjected to in-plane and out-of-plane loads, as such delivering experimental evidence that such integrated system can be directly used in construction and contribute towards the reduction of CO _2_ emissions in Europe and worldwide.

## Ethics and consent

Ethical approval and consent were not required.

## Data Availability

Zenodo: Out-of-plane performance of structurally and energy retrofitted masonry walls: Geopolymer versus cement-based textile-reinforced mortar combined with thermal insulation. https://doi.org/10.5281/zenodo.8415343 ( Cholostiakow *et al.,* 2023c) This project contains the following underlying data: Figure 1.jpg Figure 2.jpg Figure 3.jpg Figure 4.jpg Figure 5.jpg Figure 6.jpg Figure 7.jpg Figure 8.jpg Figure 8 - Load-deflection curves.csv Figure 9.jpg Figure 9 - Barcharts.csv Figure 10.jpg Figure 11.jpg Table 1 - Test specimens and configurations.csv Table 2 - Mechanical properties of the textiles.csv Table 3 - Properties of the binders.csv Table 4 - Main test results.csv Data are available under the terms of the Creative Commons Attribution 4.0 International license (CC-BY 4.0).
