# Supplementary material for: Out-of-plane performance of structurally and energy retrofitted masonry walls: geopolymer versus cement-based textile-reinforced mortar combined with thermal insulation
Source: Open Res Eur. 2023 Oct 30;3:186. [Version 1] doi: 10.12688/openreseurope.16724.1 (PMC10864824; doi:10.12688/openreseurope.16724.1)
Supplement: Peer review history

# Reviewer report: Reviewer response for version 1

**DOI:** 10.21956/openreseurope.18063.r37001

**Published:** 2024-02-20

**Authors:** Veerabhadragouda B Patil

**Affiliations:** 1 University of Pardubice, Pardubice, Czech Republic

The authors made good efforts to cover all literature and recent articles with precise explanations of research and explained their findings in the article entitled “Out-of-plane performance of structurally and energy retrofitted masonry walls: geopolymer versus cement-based textile-reinforced mortar combined with thermal insulation.” However, their minor revisions should be as follows:

1. Abstract: The last two lines needed to revise what exactly was needed and mention which combination is more replicable for traditional concrete.
2. Introduction: if possible, update with some recent literature data for comparative descriptions:

1. Clean Techn Environ Policy 23, 1701–1713 (2021). https://doi.org/10.1007/s10098-021-02085-0
2. Construction and Building Materials, Volume 400, 12 October 2023, 132869, https://doi.org/10.1016/j.conbuildmat.2023.132869
3. Case Studies in Construction Materials, Volume 18, July 2023, e02133, https://doi.org/10.1016/j.cscm.2023.e02133
4. Construction and Building Materials, Volume 371, 31 March 2023, 130688, https://doi.org/10.1016/j.conbuildmat.2023.130688

1. Some places use synonyms for the first time and carry them forward through the manuscript without mentioning the full form of it, If possible, authors can include a list of synonyms with full forms; it will be better for readers to go flow of the manuscript.
2. The authors needed to explain why there is variation in performance with geopolymer binding chemistry behind it (Refer Case Studies in Construction Materials, Volume 18, July 2023, e02133, https://doi.org/10.1016/j.cscm.2023.e02133).
3. Also, the results need to be produced in graphical form, which helps to compare results and helps to readers easily understand the output.
4. Figures 8, 9, 10, 11 well represented; if possible, key changes should be included below the schematic pictures. This will benefit readers.
5. The discussion part needs to expand with key point subheadings; it will be better.
6. Please explain why glass textiles perform so poorly.
7. Rewrite conclusion part with key points from best performance one to least one.
8. Finally, give practical, real-time applications of successful compositions with reasons.

Is the study design appropriate and does the work have academic merit?

Yes

Is the work clearly and accurately presented and does it cite the current literature?

Yes

If applicable, is the statistical analysis and its interpretation appropriate?

I cannot comment. A qualified statistician is required.

Are all the source data underlying the results available to ensure full reproducibility?

Yes

Are the conclusions drawn adequately supported by the results?

Yes

Are sufficient details of methods and analysis provided to allow replication by others?

Yes

Reviewer Expertise:

Molecular interactions between the heterogeneous elements of geopolymer concrete

I confirm that I have read this submission and believe that I have an appropriate level of expertise to confirm that it is of an acceptable scientific standard, however I have significant reservations, as outlined above.

# Reviewer report: Reviewer response for version 1

**DOI:** 10.21956/openreseurope.18063.r36072

**Published:** 2024-02-13

**Authors:** Dionysios Bournas

**Affiliations:** 1 European Commission, Ispra, Italy

This paper investigates the out-of-plane behavior of masonry walls with a composite system designed for both structural strengthening and energy efficiency improvements. Various configurations of the composite system, which include either thermal insulation panels directly adhered to the wall with subsequent layers of textile-reinforced mortar (TRM) or insulation panels sandwiched between two layers of TRM.  Beyond the layering arrangement of the retrofit materials, the research also considers two additional variables: a) the type of binder used in the mortars (cement-based versus geopolymer-based) and b) the type of textile (open mesh glass fibre versus basalt fibre). The findings underscore the mechanical advantages of combining TRM with thermal insulation over conventional TRM applications alone. Furthermore, it reveals that geopolymer-based mortars offer a performance comparable to that of standard cement-based mortars in the market.

 In conclusion, the findings reported in this paper are compelling however, it is advised that the authors revise their manuscript to address the following points:

 1)      The authors need to consider the economic and practical aspects associated with employing geopolymer mortars in actual retrofitting scenarios. Specifically, the cost per square meter of the employed system and/or geopolymer mortars available in the market should be analysed. How do these costs compare to those of traditional cement mortars? Additionally, the authors could break down the total cost of geopolymer mortars, delineating how much is attributed to the basalt fibers and the matrix component. It would be beneficial for the authors to incorporate a brief discussion on these points in their revised manuscript.

 2)      The masonry walls were retrofitted using three different configurations: i) TRM layers without insulation (REF); ii) combined structural and energy retrofitting with thermal insulation directly attached to the masonry surface followed by two TRM layers (IG2/IB2); iii) combined structural and energy retrofitting with thermal insulation between the two TRM layers (G1I1/B1I1). Regardless of whether these walls are part of an unreinforced masonry (URM) structure or serve as infill for a RC one, they are susceptible to both in-plane and out-of-plane forces. Consequently, applying thermal insulation directly to the masonry surface without adding textile reinforcement at this surface could adversely affect the wall's shear behaviour due to a lack of reinforcement, which is typically needed to counteract expected in-plane cracking. It is essential for the authors to address this concern at the outset of the manuscript, emphasising that such retrofitting not only poses a risk of creating air gaps between the insulation panel and the masonry but also may lead to an insufficient enhancement of the wall's in-plane strength.

 3)      On the results, subsection ‘Failure patterns and cracking behaviour’ it is reported:

 ‘ *On the other hand, the glass textile in GEO_G2 did not exhibit a full composite action with the GEO binder but a more local and premature type of failure; the glass textile rovings ruptured in the vicinity of the maximum bending moment showing also signs of partial slippage within the GEO matrix.*’

 The authors are invited to explain where this behaviour is attributed and provide an explanation for why it does not occur with the use of a cement-based binder.

 4)      Within the results section, under the subheading 'Performance of the geopolymer mortar,' the text states:

 ‘N *evertheless, GEO_G2 did not manage to match the capacity of its cementitious counterpart and failed in a flexural manner, prior to reaching shear capacity of the masonry.*’

 It is noted that specimen *CEM_G2* had the double strength than of *GEO_G2* , so the expression ‘ *did not manage to match’ *could be misleading.

 Moreover, it is also reported that ‘ *This evidence that this type of GEO mortar had a substantial influence on the performance of glass textiles already at the early stages of loading.*’ When comparing specimens CEM_G2 with GEO_G2 and CEM_G1l1 with GEO_G1l1, however, it is evident that this impact was also pronounced at maximum load.

Is the study design appropriate and does the work have academic merit?

Yes

Is the work clearly and accurately presented and does it cite the current literature?

Yes

If applicable, is the statistical analysis and its interpretation appropriate?

Yes

Are all the source data underlying the results available to ensure full reproducibility?

Partly

Are the conclusions drawn adequately supported by the results?

Yes

Are sufficient details of methods and analysis provided to allow replication by others?

Yes

Reviewer Expertise:

Advanced Composites (FRP, textile-based) in ConstructionRepair, Strengthening and Seismic Retrofitting of Existing StructuresIntegrated Sesmic and Energy Renovation of Exisiting BuildingsStructural Assessment of Existing StructuresReinforced and Precast Concrete Structures

I confirm that I have read this submission and believe that I have an appropriate level of expertise to confirm that it is of an acceptable scientific standard.

# Reviewer report: Reviewer response for version 1

**DOI:** 10.21956/openreseurope.18063.r36998

**Published:** 2024-02-10

**Authors:** Mohana R

**Affiliations:** 1 Mepco Schlenk Engineering College, Sivakasi, Tamilnadu, India

  Since it is an energy retrofitting walls made up of geopolymer, the cost variances between the conventional as well as geopolymer system have to be discussed in detail. Thus, it is suggested to add the cost-benefit analysis of retrofitting walls. As stated earlier, the percentage of energy saved in this work is also required to be estimated. Also, it is suggested to discuss the major key findings in abstract and conclusion part. Since the conclusion part is academically lacking, it is recommended to modify the conclusion part.

             As this project used both glass and basalt textile, whether the retrofitting wall will be more economical than the conventional one? Similarly, the cost of alkaline activators used in geopolymer construction is also very high compared to the conventional concrete. Is it possible to implement this geopolymer walls in real time practice? Kindly, clarify these statements in your research article. In addition to this, it is suggested to include the chemical composition and reaction kinetics of materials used in both the cement and geopolymer concrete. Also, the key results need to be discussed based up on the microstructural results.

Is the study design appropriate and does the work have academic merit?

Yes

Is the work clearly and accurately presented and does it cite the current literature?

Yes

If applicable, is the statistical analysis and its interpretation appropriate?

Not applicable

Are all the source data underlying the results available to ensure full reproducibility?

Partly

Are the conclusions drawn adequately supported by the results?

Partly

Are sufficient details of methods and analysis provided to allow replication by others?

Partly

Reviewer Expertise:

Geopolymer concrete

I confirm that I have read this submission and believe that I have an appropriate level of expertise to confirm that it is of an acceptable scientific standard, however I have significant reservations, as outlined above.

# Reviewer report: Reviewer response for version 1

**DOI:** 10.21956/openreseurope.18063.r36999

**Published:** 2024-01-08

**Authors:** Jitong Zhao

**Affiliations:** 1 Institute of Construction Materials, Technical University of Dresden, Dresden, Germany

The paper investigates the out-of-plane performance of masonry walls upgraded with an integrated structural and energy retrofitting system. Configurations include thermal insulation boards with TRM in different layouts and with cement and geopolymer coatings. The results emphasizes the potential of a composite system using adhesive mortars, textiles, and EPS board for improving structural and energy performance. The conclusion could be more concise, focusing on key findings and their implications. Further exploration of the unsuccessful geopolymer-glass textile combination would be insightful. The paper reports new knowledge and is of interest to the scientific community. However, some revisions should be made according to the following comments/questions:

 Detailed comments:

 1. In the introduction, state of the art mineral coating technology for construction should also involve synonyms like FRCM, MCF and corresponding literature, e.g., ^1^, ^2^, ^3^.

 2. the methodology of the production of Mortars made with geopolymer and cement binders should be given in details. Also the information of supplier on the raw materials applied. Please give the number of samples for each test? The polypropylene fibres applied in geopolymer and cement mortars were same?

 3. Geopolymer with glass textiles exhibited poor performance. Could author elaborate more on the reasons for this and potential improvements could enhance the paper.

 4. Figures 8, 10, 11, I suggest also check and use stress-strain curves.

 5. The paper frequently refers to previous studies (e.g., Cholostiakow et al., 2023a), and it might be helpful to include a comprehensive list of references for readers interested in further exploration.

 6. The paper could benefit from a section providing recommendations for practical applications based on the findings. This could include guidelines for selecting suitable textiles, binders, and retrofitting layouts.

 7. The results mention instances of debonding of insulation boards and incompatibility issues. Further discussion on the factors contributing to debonding and potential solutions could be helpful.

Is the study design appropriate and does the work have academic merit?

Yes

Is the work clearly and accurately presented and does it cite the current literature?

Partly

If applicable, is the statistical analysis and its interpretation appropriate?

Yes

Are all the source data underlying the results available to ensure full reproducibility?

Partly

Are the conclusions drawn adequately supported by the results?

Partly

Are sufficient details of methods and analysis provided to allow replication by others?

Partly

Reviewer Expertise:

Thank you so much for the invitation! My research focuses on fiber-reinforced polymer applications in construction, mineral impregnation techniques for fibers, low-carbon cementitious materials such as geopolymers and magnesium binders, and additive manufacturing.

I confirm that I have read this submission and believe that I have an appropriate level of expertise to confirm that it is of an acceptable scientific standard, however I have significant reservations, as outlined above.

## References

[1] : Failure modes in FRCM systems with dry and pre-impregnated carbon yarns: Experiments and modeling. *Composites Part B: Engineering* .2018;140: 10.1016/j.compositesb.2017.12.024 57-67 10.1016/j.compositesb.2017.12.024

[2] : Development and testing of fast curing, mineral-impregnated carbon fiber (MCF) reinforcements based on metakaolin-made geopolymers. *Cement and Concrete Composites* .2021;116: 10.1016/j.cemconcomp.2020.103898 10.1016/j.cemconcomp.2020.103898

[3] : Mineral-impregnated carbon fiber composites as novel reinforcement for concrete construction: Material and automation perspectives. *Automation in Construction* .2020;110: 10.1016/j.autcon.2019.103002 10.1016/j.autcon.2019.103002
